# The Treatment of Heart Failure-Related Symptoms with Ivabradine in a Case with Peripartum Cardiomyopathy

**Published:** 2013-03-15

**Authors:** Serafettin Demir, Mucahit Tufenk, Zeynep Karakaya, Rabia Akilli, Mehmet Kanadas

**Affiliations:** 1 Department of Cardiology, Adana State Hospital, Adana, Turkey; 2 Department of Cardiology, Kiziltepe State Hospital, Mardin, Turkey; 3 Department of Emergency Medicine, Adana State Hospital, Adana, Turkey; 4 Department of Cardiology, School of Medicine, Cukurova University, Adana, Turkey

**Keywords:** Heart Failure, Ivabradine, Peripartum Cardiomyopathy

## Abstract

**Abstract:**

Peripartum cardiomyopathy is a form of dilated cardiomyopathy that is defined as deterioration in cardiac function presenting typically between the last month of pregnancy and up to five months postpartum. As with other forms of dilated cardiomyopathy, PPCM involves systolic dysfunction of the heart with a decrease of the left ventricular ejection fraction with associated congestive heart failure. In heart failure sinus tachycardia is a poor prognostic factor and the common symptom. In this paper, we presented a case treated with ivabradine which provided additional benefit in patient with acute heart failure.

## 1. Introduction

Peripartum cardiomyopathy (PPCM) is a form of dilated cardiomyopathy that is defined as deterioration in cardiac function presenting typically between the last month of pregnancy and up to five months postpartum. As with other forms of dilated cardiomyopathy, PPCM involves systolic dysfunction of the heart with a decrease of the left ventricular ejection fraction (EF) with associated congestive heart failure. In heart failure sinus tachycardia is a poor prognostic factor and the common symptom. In this paper we presented a case, using ivabradine which provided additional benefit in patient with acute heart failure.

Peripartum cardiomyopathy (PPCM) is a rarely encountered disease with high mortalities in peripartum period. The incidence of the disease differs from 1/1300 to 1/15000. Multiparity, advanced maternal age, gestational hypertension (pregnancy-induced hypertension), preeclampsia and black race are among the risk factors of the disease (1). Although PPCM and idiopathic dilated cardiomyopathy (IDC) have similar clinical and hemodynamic features, they differ in terms of histological characteristics and prognosis. While IDC has a rather slow clinical course, PPCM has a fast clinical deterioration with high mortality. The aetiology of the disease is still unknown. However, myocarditis, abnormal immune response to pregnancy, inappropriate adaptation due to increased hemodynamic stress in pregnancy, cytokines triggered by stress, viral infections and prolonged tocolysis are suspected (2).

Sinus tachycardia implicates a poor prognosis and the common symptom in heart failure. Conventional drugs such as beta blockers and calcium antagonist that used to reduce heart rate worsens the symptoms of heart failure in acute stage. Ivabradine, selective *I*f channel blocker, is a new drug for using to reduce heart rate without hemodynamic negative effect. In this paper we presented a case treated with ivabradine that provides additional benefit in patients with acute heart failure.

## 2. Case Report

A 36-year old housewife referred to our hospital with a complaint of dyspnea starting within the 36th week of its pregnancy and continued for 2 months. Dyspnea had been significant for recent weeks even with ordinary housekeeping activities. Patient started to sleep with two pillows and according to her, the dyspnea increased while she was lying on her back. Swellings in her legs occurred and the severity of dyspnea increased gradually. The patient was referred to our hospital due to a heart failure related to pregnancy period. 

**Figure 1:**
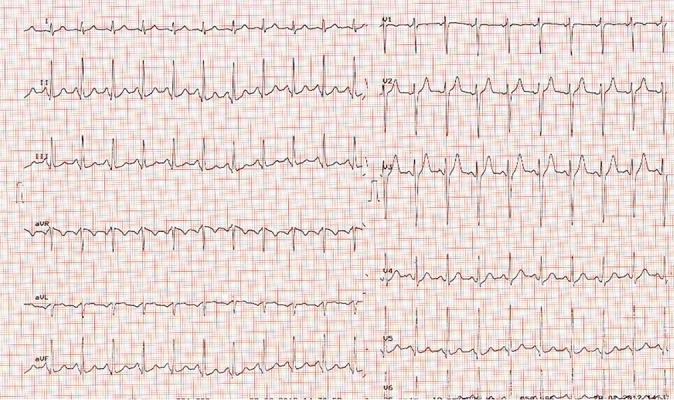
The Electrocardiogram of Patients before Administration of Ivabradine

Patient was hospitalized in our cardiology department. In her physical examination, orthopnea and bilateral basal rales were detected. Other symptoms detected included venous distension in her neck, hepatojugular reflux, S3 (third heart sound) and pitting, ++/++ pretibial edema. Blood pressure was 105/75 and patient had a tachycardia with 119 bpm. As shown in Figure 1 sinus tachycardia was found in her derived 12- lead electrocardiogram (ECG). Her echocardiography also revealed diffused wall motion disorder, biventricular dilatation, systolic dysfunction, diastolic left ventricular dysfunction and mild pericardial effusion. Ejection fraction was determined to be 32% via Simpson’s technique.

The patient was then treated with ACE inhibitors, parenteral furosemide, spironolactone, low molecular weight heparin, digital, carnitine, oxygen and proton pump inhibitor. Since the patient’s tachycardia was associated with cardiac failure and no improvement was observed in the failure symptoms, she was administered levosimendan through the parenteral route. In her physical examination following the treatment with levosimendan, the rales in her lungs were observed to diminish, although the S3, orthopnea and tachycardia were continuing during the auscultation. On the fifth day of follow-up, metoprolol was added to the treatment of the patient with dosage of 12.5 mg per day because of ongoing resting sinus tachycardia (103 bpm, Figure 1), despite digital treatment. Nevertheless, patient was not able to tolerate beta-blocker due to her increasing dyspnea. A selective If blocker, Ivabradine, 5 mg twice daily, was thereupon added to the treatment of the patient.

**Figure 2:**
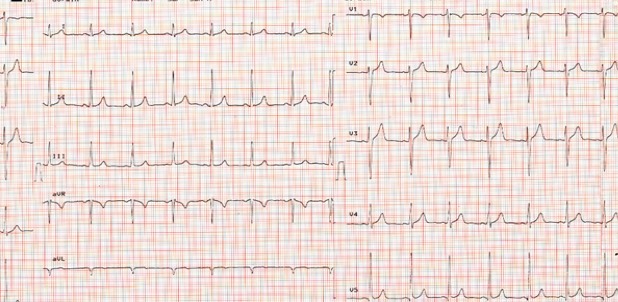
The Electrocardiogram of Patients following Ivabradine Treatment

After including Ivabradine in the treatment, the tachycardia of the patient was ameliorated with heart rate falling to 80 bpm within 12 hours (Figure 2). On the seventh day of the follow-up, orthopnea of the patient was treated without any additional medical intervention. In her physical examination, S3 was obliterated and dyspnea was significantly improved. After Ivabradine administration, there was a remarkable increase in patient's physical activity, a condition not present previously. The patient's ECG showed a normal heart rate and there were no differences detected in blood urea nitrogen, creatine, blood sodium and potassium, hematocrit levels of the patient who had undergone routine lab tests and ECG follow-up. The Ivabradine treatment was not associated with any adverse effects within 5 days of follow-up.

## 3. Discussion

Researchers showed that e heart rate is an independent indicator of mortality in heart failure (3). Tachycardia is associated with increased sympathetic activity and decreased vagal activity. High heart rate shortens longevity at the time of resting. In addition to this, tachycardia in resting is a potent and an independent predictor of cardiovascular morbidity and mortality. In the course of tachycardia, the period required for myocardial perfusion is reduced and diastolic and systolic functions are deteriorated progressively depending on the shortening of diastole time. Thus, not only heart rate but also myocardial oxygen consumption of the patients with heart failure should be diminished. Beta blockers reduce the demand for oxygen by decreasing heart rate and pressure. However, one of the five patients could not respond to or tolerate beta blockers (4). Our patient was not able to tolerate beta blockers as the heart failure was at decompensated level. Benefit from digoxin is observed at most in patients, who belong to the class of NYHA III and IV. In such cases, the response of the circulation to digital therapy is at most characterized by a decrease in venous pressure, reduced ventricular filling pressure, and an increase in cardiac flow. Heart rate slows down and ejection fraction is liable to increase. It is thought that these effects depend on cardiac baroreceptor sensitization associated with Na-K ATPase inhibition of vagal afferent nerve fibers, and therefore on reduction of sympathetic outflow released from central nervous system (5). Furthermore, sodium delivery to distal tubules increases with Na-K inhibition in kidney tubules with consequent release of renin from kidneys. All these observations gave rise to the thought that along with its positive inotropic effect, digitalin administration in the treatment of heart failure is efficient, since it could repress neurohumoral response. While myocardial oxygen consumption increases as the contractility rises in a normal heart, oxygen consumption decreases as a result of the fall in heart rate, a decrease in ventricular wall tension and a downsizing in heart dimensions in the treatment of heart failure with digoxin. The digitalin was added to our patient’s treatment by taking these benefits into consideration, yet monitoring did not reveal any sufficient response to digitalin therapy. As our patient had a heart failure, calcium canal blockers were not included in therapy.

Being a selective *I*f channel blocker, Ivabradine decreases heart rate by reducing sinus node automaticity. Researchers showed that Ivabradin decreases myocardial oxygen demand and anginal symptoms by reducing heart rate in the case of stable angina pectoris (6). Besides, it is stated that Ivabradine reduces heart rate without increasing congestive failure in patients, whose left ventricle function is distorted and have beta-blocker intolerance or a contraindication to beta blockers. As it has no negative inotropic effect, Ivabradine is advantageous in the treatment of patients with left ventricular dysfunction, especially in decompensated heart failure, compared with beta-blockers. In this PPCM case which also included a decompensated heart failure, we considered sinus tachycardia both as a symptom and a pathophysiological state, which required treatment. Since patient had intolerance to beta blockers, Ivabradine, an *I*f channel blocker with negative inotropic effect was preferred to control tachycardia.

 On the third day of Ivabradine administration, patient was discharged from the hospital with a remarkable amelioration in the symptoms of congestive failure.

 As a result, this case showed that Ivabradine could be administered to ameliorate the symptoms and signs of heart failure in a tachycardic dilated cardiomyopathy with decompansated congestive failure. Ivabradine could be beneficial in beta blocker intolerance and tachycardic decompensated congestive heart failure.

## References

[1] Cunningham FG, Pritchard JA, Hankins GD, Anderson PL, Lucas MJ, Armstrong KF (1986). Peripartum heart failure: idiopathic cardiomyopathy or compounding cardiovascular events?. Obstet Gynecol..

[2] Pearson GD, Veille JC, Rahimtoola S, Hsia J, Oakley CM, Hosenpud JD (2000). Peripartum cardiomyopathy: National Heart, Lung, and Blood Institute and Office of Rare Diseases (National Institutes of Health) workshop recommendations and review.. JAMA..

[3] Purcell H (1999). Heart rate as a therapeutic target in ischaemic heart disease.. Eur Heart J..

[4] Butler J, Young JB, Abraham WT, Bourge RC, Adams KF, Clare R (2006). Beta-blocker use and outcomes among hospitalized heart failure patients.. J Am Coll Cardiol..

[5] Gheorghiade M, Adams KF, Jr., Colucci WS (2004). Digoxin in the management of cardiovascular disorders.. Circulation..

[6] Borer JS, Fox K, Jaillon P, Lerebours G (2003). Antianginal and antiischemic effects of ivabradine, an I(f) inhibitor, in stable angina: a randomized, double-blind, multicentered, placebo-controlled trial.. Circulation..

